# Utilizing machine learning algorithms to predict subject genetic mutation class from in silico models of neuronal networks

**DOI:** 10.1186/s12911-022-02038-7

**Published:** 2022-11-09

**Authors:** Gavin T. Kress, Fion Chan, Claudia A. Garcia, Warren S. Merrifield

**Affiliations:** 1 NeuroDetect, Cambridge, MA USA; 2 University of California, Irvine, Irvine, CA USA

**Keywords:** Machine learning, Neural network, Support vector machine, Gaussian naïve bayes, Decision tree, Gradient boosting decision tree, Epilepsy, Dravet syndrome, SCN1A, In silico, BRIAN2, Diagnosis, Genomics, Electrophysiology, Microelectrode array

## Abstract

**Background:**

Epilepsy is the fourth-most common neurological disorder, affecting an estimated 50 million patients globally. Nearly 40% of patients have uncontrolled seizures yet incur 80% of the cost. Anti-epileptic drugs commonly result in resistance and reversion to uncontrolled drug-resistant epilepsy and are often associated with significant adverse effects. This has led to a trial-and-error system in which physicians spend months to years attempting to identify the optimal therapeutic approach.

**Objective:**

To investigate the potential clinical utility from the context of optimal therapeutic prediction of characterizing cellular electrophysiology. It is well-established that genomic data alone can sometimes be predictive of effective therapeutic approach. Thus, to assess the predictive power of electrophysiological data, machine learning strategies are implemented to predict a subject’s genetically defined class in an in silico model using brief electrophysiological recordings obtained from simulated neuronal networks.

**Methods:**

A dynamic network of isogenic neurons is modeled in silico for 1-s for 228 dynamically modeled patients falling into one of three categories: healthy, general sodium channel gain of function, or inhibitory sodium channel loss of function. Data from previous studies investigating the electrophysiological and cellular properties of neurons in vitro are used to define the parameters governing said models. Ninety-two electrophysiological features defining the nature and consistency of network connectivity, activity, waveform shape, and complexity are extracted for each patient network and t-tests are used for feature selection for the following machine learning algorithms: Neural Network, Support Vector Machine, Gaussian Naïve Bayes Classifier, Decision Tree, and Gradient Boosting Decision Tree. Finally, their performance in accurately predicting which genetic category the subjects fall under is assessed.

**Results:**

Several machine learning algorithms excel in using electrophysiological data from isogenic neurons to accurately predict genetic class with a Gaussian Naïve Bayes Classifier predicting healthy, gain of function, and overall, with the best accuracy, area under the curve, and F1. The Gradient Boosting Decision Tree performs the best for loss of function models indicated by the same metrics.

**Conclusions:**

It is possible for machine learning algorithms to use electrophysiological data to predict clinically valuable metrics such as optimal therapeutic approach, especially when combining several models.

**Supplementary Information:**

The online version contains supplementary material available at 10.1186/s12911-022-02038-7.

## Introduction

Epilepsy is a highly prevalent neurological disorder which affects all age groups. The majority of patients with epilepsy will not go into remission from their first antiepileptic drug (AED) with up to 40% percent of patients experiencing seizures that are not controlled by medication [1]. Not only does this potentially impart permanent injury, but it accounts for 80% of the cost of epilepsy in the United States. Moreover, more than 66% of patients with unmanaged seizures are considered to have refractory or drug resistant epilepsy (DRE) [2]. This implies that patients that initially go into remission have a high likelihood of the efficacy of the therapy diminishing over time to the point of not sufficiently managing epileptic activity at all.

Forty percent of patients experience intense adverse effects that significantly impact quality of life and contribute to treatment failure. These can occur for a variety of reasons ranging from the inability to metabolize the therapeutic to a complex combination of specific genomic, proteomic, metabolomic, transcriptomic and manifestly electrophysiological (EP) characteristics [3]. These adverse effects include fatigue, memory loss, gastrointestinal disturbances, cardiovascular disturbances, vision problems, nausea, ataxia, cephalgia, insomnia, depression, and much more. The reported side effects are not uncommon, with most of the individual adverse effects occurring in 10–40% of patients, and up to 88% of patients experiencing at least one [4].

The current standard of care, when a patient is not responding to a particular AED monotherapy or polytherapy, or the adverse effects are unmanageable and the quality of life is significantly impacted, is to proceed through a trial-and-error system to find a monotherapy or polytherapy that is both effective and has manageable adverse effects. This process can take years, cost hundreds of thousands of dollars, and impart irreparable harm and suffering to the patient [2]. Therefore, there exists a large interest in novel strategies to predict how a patient will respond to a particular medication a priori. While some progress has been made regarding the use of genomic information to predict treatment efficacy, it is far from capable of predicting the ideal therapeutic approach in most patients [5]. One contributing element to the lack of predictive power in pharmacogenomics is the myriad of other factors that dynamically affect the electrophysiology of patient neurons. These factors include metabolomics, transcriptomics, proteomics, epigenetic variables, and many more.

Given the number of variables that have the potential to affect patient electrophysiology and therapeutic efficacy, it is implausible to expect that a single physician should be able to strategically synthesize them and derive the optimal therapeutic approach. Therefore, there has been considerable interest in implementing machine learning (ML) algorithms to guide patient classification.

In addition to implementing novel strategies to cohesively consider the information relevant to predicting therapeutic efficacy, there is also a growing interest in identifying novel biomarkers and parameters that are predictive of therapeutic efficacy. Recently, variables extracted from patient cellular electrophysiology have shown great promise in their predictive power regarding a number of neurological conditions. For example, dynamical electrical complexity has been shown to be reduced in neurons isogenic to patients with autism spectrum disorders and epilepsy has been shown to be related to theta band network connectivity as well as interictal spike and paroxysmal depolarization shift frequency [6–9]. These results implicate the strategy of using patient derived induced pluripotent stem cells (iPSCs) to create isogenic neuron and/or glial cells which can be cultured or co-cultured on microelectrode arrays (MEA) or nanoelectrode arrays (NEA) to record EP activity and extract relevant biomarkers. Recent improvements in the speed and efficiency with which skin associated fibroblasts and peripheral blood monoclonal cells (PBMCs) can be induced into iPSCs [10, 11], as well as improvements in speed and yield of neurons differentiated from iPSCs [12, 13], are bringing this approach closer to clinical feasibility at a large scale. Moreover, demand for reagents theoretically exert market forces to scale the production of said reagents, driving down the price, and further incentivizing this type of testing; with better predictive biomarkers, it may be possible to identify an effective therapeutic strategy at an earlier stage, thereby significantly reducing the healthcare cost while improving patient prognoses [2].

It has been well-established that genetic variables are effective at coarsely predicting which therapies may be more effective in some cases [5]. It is of current interest to assess the ability of electrophysiology to have similar predictive power. Recent studies have shown that ML algorithms can be used to extract features from electrophysiological data [14]. Furthermore, it has been shown that electrophysiological data can be utilized in isolation by machine learning algorithms to identify pathologic states or perturbations [15, 16]. Much of the current work in the field is focused on cardiac pathologies and typically investigate only one machine learning method such as a support vector machine or Bayesian supervised learning method. Therefore, it is not only of interest to explore these methods in neuronal applications but also to assess the capability of various different statistical and machine learning methods to synthesize a large quantity of relevant information to make such predictions about the state of neuronal cultures.

Ideally, a large-scale clinical study involving a moderately variable group of patients with differently originating and manifesting epilepsy would be conducted to answer these questions. In such study, one could extract skin associated fibroblasts or PBMCs to induce to iPSCs and further differentiate to isogenic neurons and glial cells. These cells could be co-cultured on an MEA to record EP activity and further processing of the data would extract the relevant EP variables. These variables would then be subjected to statistical analysis to determine if they are predictive of actual patient treatment response. Although such a study involves subjecting patients to minimally invasive, low-risk procedures, these procedures carry inherent risk, nonetheless. This study design would also take a considerable amount of time and resources to complete.

Therefore, it is important to perform pre-validation studies to confirm the scientific promise of conducting such an extensive study. Thus, the goal of this exploration is to assess the ability of EP variables to predict therapeutic response in an efficient manner. Since genetic mutation status has been shown to predict therapeutic efficacy to some degree [5] and because certain genetic mutations provoke alterations in the presentation of epileptic disease on an EP level, it follows that genetic based differences in EP presentation should also have predictive power for identifying the more effective treatment strategy for the individual. Moreover, from an information theory perspective, something that is predictive of genomic mutation status should also have predictive power for therapeutic efficacy given genomic mutation status is, of itself, predictive of therapeutic efficacy. This point is important because this study utilizes in silico modeling of neuronal cultures and modeling of disease status defined by a unique genetic mutation, which exerts a very well-defined action that is much more direct and therefore reliable than in silico modeling of a particular therapy [17].

In order to accomplish this, a small-scale dynamic network of isogenic neurons is modeled in silico for 1 s for 228 dynamically modeled patients. There are thousands of unique epileptogenic genetic mutations, many of which are voltage-gated sodium channel (VGSC) mutations. These mutations are usually either global gain of function (GoF) or loss of function (LoF) in VGSC isoforms highly selectively expressed in inhibitory neurons, both of which cases result in increased network excitability [18]. Thus in the current in silico model, patients fall into one of three categories: healthy, general sodium channel GoF mutation (GoF), or inhibitory sodium channel LoF. Following this, a number of simple and advanced EP parameters are extracted for each patient. Using statistical tests to determine which variables serve as inputs to ML algorithms, several different ML algorithms are tested to assess their relative performance in predicting which genetic category the subject falls under.

## Methods

### In Silico* modeling*

In order to recapitulate the most important features of neuronal networks in a dynamic and customizable manner, the BRIAN 2 Python library was utilized. Data used to determine parameters that define the following model were obtained from Stimberg et al. [17], BRIAN 2 documentation, and Hodgkin-Huxley [19], and summarized in Table 1. A modified version of Hodgkin-Huxley [19] dynamics were used to govern the membrane voltage response with the system of differential Eqs. 1–6.

1 $$\frac{dV}{{dt}} = \frac{{g_{l} \left( {E_{l} - V} \right) + g_{Na} m^{3} h\left( {E_{Na} - V} \right) + g_{k} n^{4} \left( {E_{K} - V} \right)}}{{C_{m} }}$$

2 $$\frac{dm}{{dt}} = \frac{{0.32\left( {13 - V + VT} \right)}}{{e^{{\frac{13 - V + VT}{4}}} - 1}}\left( {1 - m} \right) - \frac{{0.28\left( {V - VT - 40} \right)}}{{e^{{\frac{V - VT - 40}{5}}} - 1}}m$$

3 $$\frac{dn}{{dt}} = \frac{{0.032\left( {15 - V + VT} \right)}}{{e^{{\frac{15 - V + VT}{5}}} - 1}}\left( {1 - n} \right) - 0.5e^{{\frac{10 - V + VT}{{40}}}} n$$

4 $$\frac{dh}{{dt}} = 0.128e^{{\frac{17 - V + VT}{{18}}}} \left( {1 - h} \right) - \frac{4}{{1 + e^{{\frac{40 - V + VT}{5}}} }}h$$

5 $$\frac{{dg_{Na} }}{dt} = \frac{{\left( {g_{Na, 0} - g_{Na} } \right)}}{{\tau_{Na} }}$$

6 $$\frac{{dg_{K} }}{dt} = \frac{{\left( {g_{K, 0} - g_{K} } \right)}}{{\tau_{K} }}$$

**Table 1:** Summary of parameters adapted from Stimberg et al. (2017), (17) BRIAN 2 documentation, and Hodgkin-Huxley (1952) (19) for healthy patients in the in silico simulation of a 2D neuronal network

Parameter	Value	Units
τ_K_	6.00	ms
τ_Na_	6.00	ms
g_l_	50.00	µS cm^−2^ Area
g_Na,0_	100.00	mS cm^−2^ Area
g_K,0_	30.00	mS cm^−2^ Area
Ω_f_	3.33	s^−1^
Ω_d_	2.00	s^−1^
U_0_	0.6	

With V representing the membrane voltage in mV, n, m, and h are dimensionless values between 0 and 1 representing the proportion of the n, m, and h gates open in their respective voltage-gated ion channels. The values g_l_, g_k_, and g_Na_ represent the leak, potassium, and sodium conductances in siemens (S), respectively, and the corresponding subscripted E values are the leak, potassium, and sodium Nernst potentials in mV. VT, g_Na,0_, and g_K,0_ are the threshold potential in mV and resting sodium and potassium membrane conductances in S. Finally, C_m_ is the membrane capacitance in Farads and $\tau_{Na}$ and $\tau_{K}$ are the time constants associated with the clearance of ligands opening ligand-gated sodium and potassium channels in ms, respectively.

For simplicity, only sodium and potassium channels were explicitly modeled, with the rest of the dynamics surmised and allocated to the leak variables. The modified opening and closing coefficients for the gating dynamics are from the BRIAN 2 documentation to optimize physiologic recapitulation under current injection-like conditions with a defined threshold voltage, which in this case was defined as − 63 mV. Passing the threshold defines a firing event, and the refractory parameter is set to the same threshold such that a spike is not counted more than once; see the BRIAN 2 documentation for more information about threshold and refractory configurations in current injection Hodgkin-Huxley type models. The method used to solve the system of differential equations was exponential Euler in time steps of 0.1 ms.

Neurons were initialized on a small scale in silico 2-dimensional array, which is 20 electrodes by 10 electrodes with 10 µm electrode spacing. Two-hundred neurons were centered on each of the 200 electrodes, 132 of which were excitatory and 68 inhibitory. This is consistent with estimates of excitatory to inhibitory neuron ratios [20, 21]. Each neuron was placed randomly on the array using the random module in Python and initialized with a random size uniformly distributed between 10,000 and 30,000 µm^2^, consistent with literature ranges [20]. Additionally, each neuron was given a random starting voltage uniformly distributed between the leak Nernst potential and the threshold potential, as well as a random initial sodium and potassium membrane conductance governed by Eq. 7.

7 $$g_{i} = \left( {\eta + 1} \right)w_{i} A$$

Here, i corresponds to sodium or potassium, w_i_ is the synaptic weight of excitatory and inhibitory connections, respectively, and $\eta$ is a random number between 0 and 1. Each of the neurons on the array are randomly connected based on a probability defined by the 2-D Gaussian distribution in Eqs. 8 and 9, with the condition that a neuron cannot be connected to itself.

8 $$P_{E} = {\text{E exp}}\left[ {\frac{{\left( {x_{pre} - x_{post} } \right)^{2} + \left( {y_{pre} - y_{post} } \right)^{2} }}{{2SD_{E}^{2} }}} \right]$$

9 $$P_{I} = I {\text{exp}}\left[ {\frac{{\left( {x_{pre} - x_{post} } \right)^{2} + \left( {y_{pre} - y_{post} } \right)^{2} }}{{2SD_{I}^{2} }}} \right]$$

In these equations, E and I represent the amplitude of the gaussian distribution for excitatory and inhibitory presynaptic neurons, respectively. The variables x and y indicate the location of the neuron (presynaptic or postsynaptic indicated by the subscript), and the standard deviation of the Gaussian Distribution is given by SD. The probabilities and standard deviations have subscripts indicating the different values for inhibitory and excitatory presynaptic neurons.

Synaptic dynamics were governed by the phenomenological description of synaptic short-term plasticity by Tsodyks and Markram [22, 23]. This description is partly expressed in Eqs. 10 and 11.

10 $$\frac{{du_{S} }}{dt} = - {\Omega }_{f} u_{S}$$

11 $$\frac{{dx_{S} }}{dt} = {\Omega }_{d} \left( {1 - x_{S} } \right)$$

where $u_{S}$ relates to the docked neurotransmitter resources for synaptic exocytosis and $x_{S}$ is the fraction of total neurotransmitter available for release [17]. It is evident that between firing events $u_{S}$ decays to 0 at a rate ${\Omega }_{f}$ and $x_{S}$ approaches 1 at rate ${\Omega }_{d}$.

When the presynaptic neuron fires, the calcium influx at the presynaptic terminal provokes a fraction $U_{0}$ of the neurotransmitter to become available for release. Following this, the proportion of the docked neurotransmitters ready for release are released ($r_{S}$), and of course, the released neurotransmitters are deducted from the fraction available for release. This is expressed in Eqs. 12–14.

12 $$u_{S} \to u_{S} + U_{0} \left( {1 - u_{S} } \right)$$

13 $$r_{S} = u_{S} x_{S}$$

14 $$x_{S} \to x_{S} - r_{S}$$

Finally, when a presynaptic neuron fires it exerts an action on all of the postsynaptic neurons which it synapses on, governed by Eqs. 15 and 16.

15 $$g_{Na} \to g_{Na} + w_{e} r_{S} R_{E}$$

16 $$g_{K} \to g_{K} + w_{i} r_{S} R_{I}$$

Here, w is the weight of the synapse indicated in Eq. 7 and r is the ratio of the synaptic weight in these equations to the initialization weight in the reference equation, which were set to 15.1 for excitatory and 9 for inhibitory to optimize recapitulation of physiological activity. The Eqs. 1–16 along with the noted parameters and connection methodology completely describe the structure of the in silico model. The leak Nernst potential was set at − 65 mV, with sodium and potassium Nernst potentials set to 50 mV and − 90 mV, respectively, which are within the ranges reported in the literature [24–29]. The excitatory and inhibitory synaptic weights, w, were set at 70 mS and 20 mS, respectively; the connection amplitudes and standard deviations for excitatory and inhibitory presynaptic neurons were 0.83 (15.5 µm) and 0.41 (12 µm), respectively. These were chosen to optimize the excitatory to inhibitory ratio for healthy patients such that activity best represented in vitro experiments.

The remaining parameters for healthy patients were adapted from Stimberg et al. [17], BRIAN 2 documentation, and Hodgkin-Huxley [19] to optimally recapitulate neuronal network activity based on the current configuration. These are summarized in Table 1.

The parameters above fully describe the healthy patient parameters. GoF patients were modeled as if they had a global Nav 1.1 mutation which leads to the adoption of open channel conductance at all times for the proportion of sodium channels of the Nav 1.1 form which ranges from 5 to 20% of all membrane sodium channels [30]. The disease neurons were modeled to express the Nav 1.1 isoform at 20%, with an open conductance that is 80-fold that of the closed channel as noted in Hodgkin and Huxley [19]. This specific mutation often results in Dravet Syndrome, a subtype of epilepsy common in younger patient populations [31]. The increase in conductance for these isoforms manifests as a global disease-state sodium leak conductance of 1680 mS cm^−2^ Area. For LoF models, it was assumed that the mutation affects a negligible proportion of sodium channel isoforms in excitatory neurons and roughly 20% of sodium channels in inhibitory neurons. The mutation was modeled to cause the sodium channel to have roughly no leak conductance, thus reducing the sodium leak conductance to 80 mS cm^−2^ Area; since this isoform was modeled as a voltage-gated ion channel, the synaptic dynamics were unchanged. Clearly, both disease models result in enhanced network excitability, which can potentially produce epileptogenicity.

**Fig. 1:**
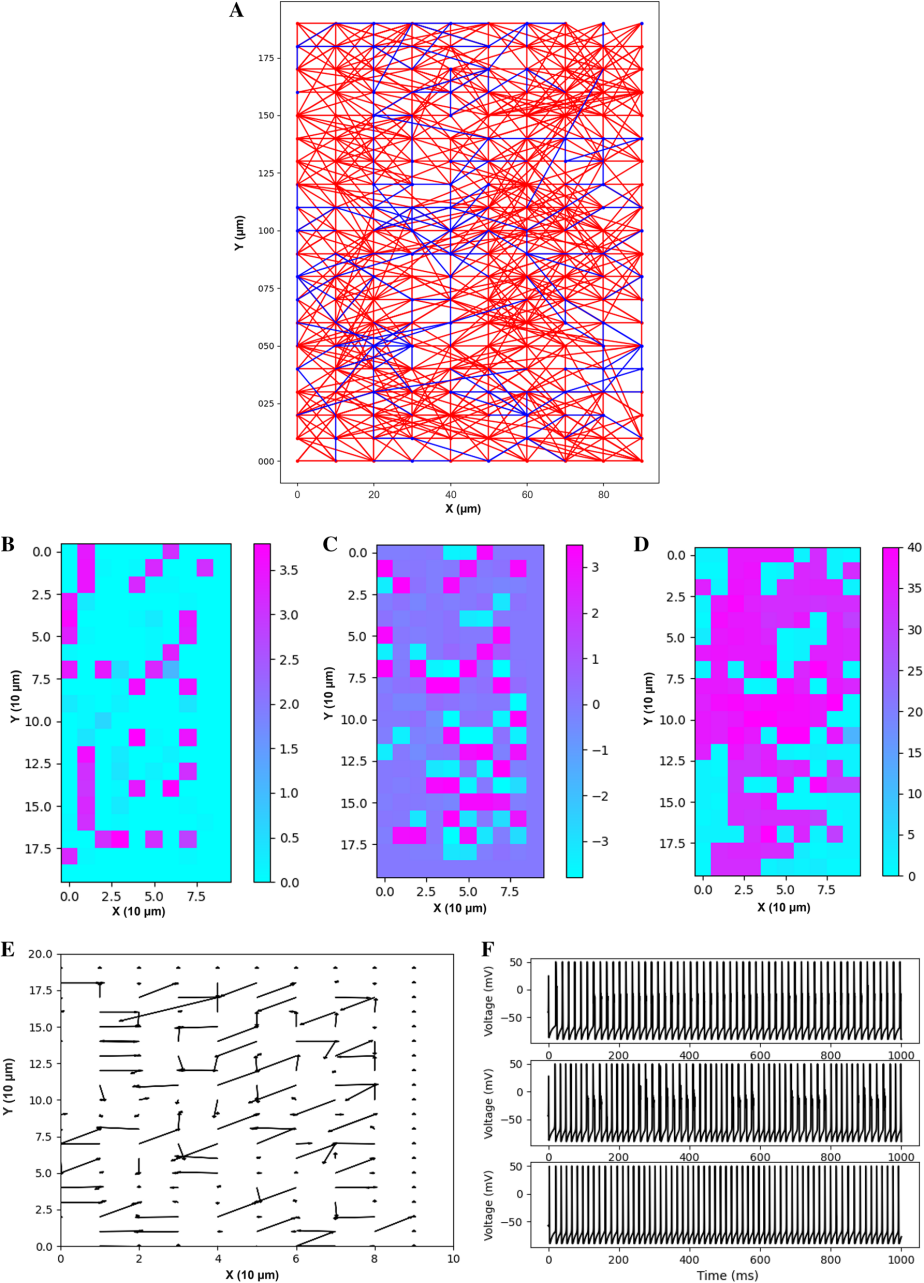

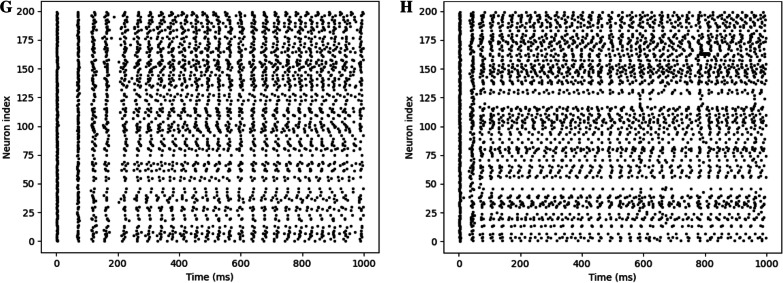
**A** Connectivity Network. Exemplar network of connections of healthy neurons modeled on a 2D array. Blue lines indicate inhibitory presynaptic neurons and red lines indicate excitatory. **B** Heat map of the x spatial gradient of the neuron frequency in Hz µm^−1^. **C** Heat map of the y spatial gradient of the neuron frequency in Hz µm^−1^. **D** Heat map of the neuron frequency in Hz. **E** Vector plot of the spatial gradient of the neuron frequency in Hz µm^−1^. **F** Three exemplar neurons’ voltage as a function of time in a healthy model. **G** Raster plot of a disease model showing synchronous (epileptiform) activity. **H** Raster plot of a healthy model showing asynchronous activity

A total of 228 subjects were modeled for 1 s of activity each: 76 Healthy, 76 GoF, and 76 LoF. Figure 1A–H show plots generated by exemplar models.

### Electrophysiological variables

For each of the neuronal network models, 92 potentially relevant EP variables were extracted. These variables mostly consist of derived variables from one of the following eight variable classes: frequency, complexity as determined by the minimum embedding dimension (MED), complexity as determined by the multiscale entropy (MSE), waveform shape indicated by paroxysmal depolarization shifts (PDS), waveform shape indicated by interictal spikes (IIS), waveform shape as indicated by line length, waveform shape as indicated by area under the curve (AUC), and functional connectivity as determined by the Transfer Shannon Entropy (TE). Moreover, the most common derived variable is a type of gradient which indicates heterogeneity and network communicability.

The frequency of each neuron on the array was measured and the following summary statistics were extracted: maximum frequency, maximum frequency spatial gradient, average frequency, and average frequency spatial gradient.

The complexity of the waveform as determined by the MED was also extracted for each neuron on the array. It has been proposed that the dynamical complexity of a neuronal voltage trace is characterized by the minimum number of dimensions it resides in as determined by the false nearest neighbor’s (FNN) algorithm [32]. Moreover, MED analyses have shown that it is associated with clinical endpoints, such as nonverbal intelligence [6]. The justification for its characterization of complexity is derived from the mathematical principle that functions embedded in higher dimensional spaces generally depend on the dimensions in which they are embedded. Therefore, voltage traces that are correctly embedded in a higher number of dimensions depend on a greater number of variables and are thus more complex in that sense. See Kennel et al. [32] for more information about how the number of dimensions is computed. In this study, the parameters used to calculate the MED are as follows: the threshold fold distance increase is 10, the threshold standard deviation is 2, and the time delay used is 50 ms. The summary statistics extracted for MED are mean, maximum, and standard deviation of the MED and MED spatial gradient.

**Fig. 2:**
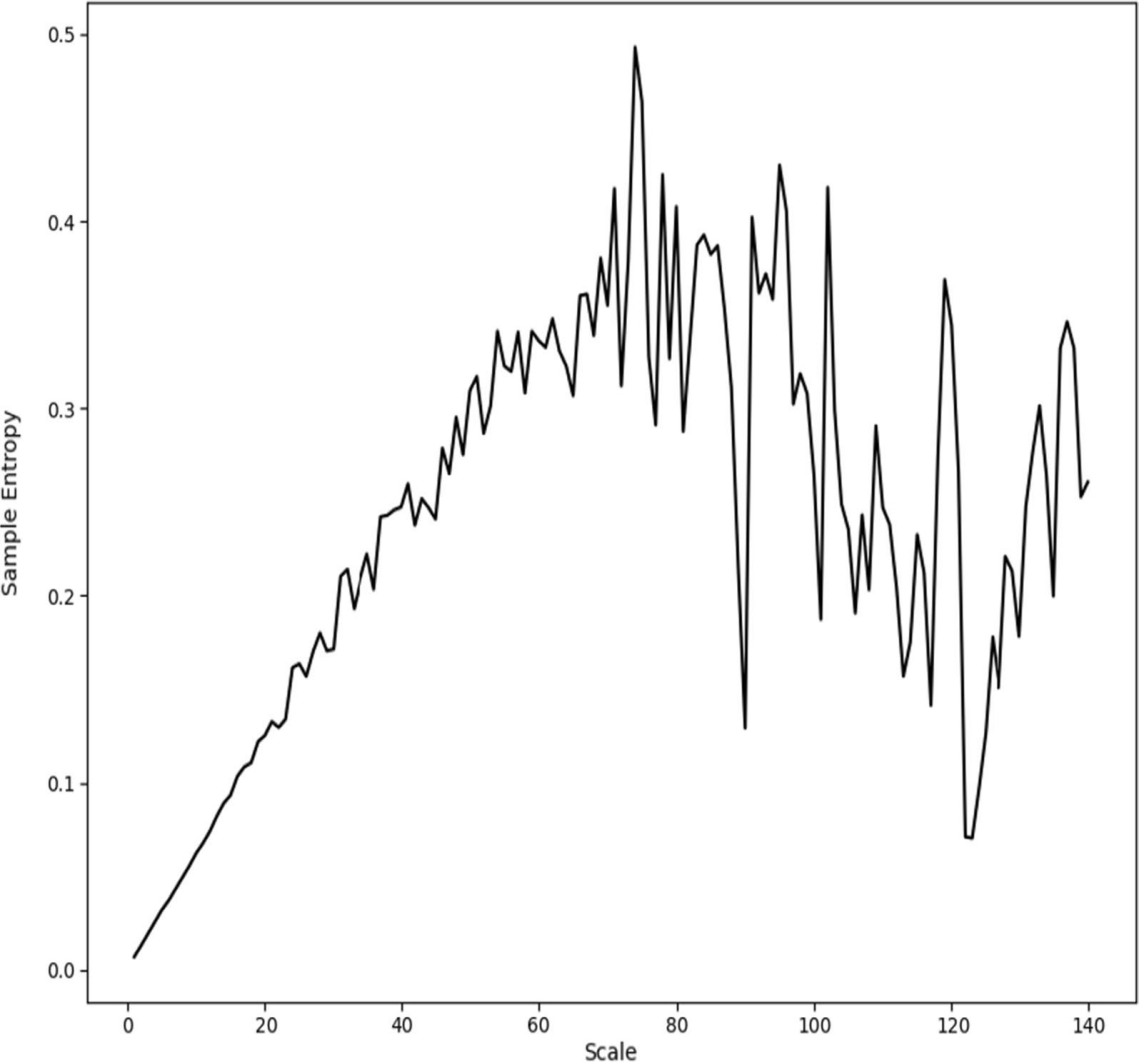
Sample entropy as a function of scale in number of time steps for an exemplar subject

Another common gauge of EP complexity is the MSE of a voltage trace [33]. MSE is a function that maps time scales to sample entropy values. The sample entropy is related to the quantity of information in a time series and thus a characterization of complexity in that way [34]. By analyzing the sample entropy at various time scales, one derives the scales in which the information in a signal resides, which is a unique and highly dynamic characteristic of a time series. Because, in this case, each neuron has a function associated with it, a large number of summary statistics can be extracted. For each function the integral, mean, and standard deviation of the function were computed, and for each of these four statistics the mean, maximum, standard deviation, mean spatial gradient, maximum spatial gradient, and spatial gradient standard deviation were extracted for a total of 18 summary statistics. A plot of the sample entropy as a function of scale for an exemplar model is shown in Fig. 2.

There are six variables extracted, characterizing the waveform shape. Two of these are derived from PDSs which are defined by action potentials that are at least 50% longer than the average action potential and two of which are also derived from the number of short IISs, defined by isolated action potentials that are less than 50% of the duration of a typical action potential. These are characteristic waveforms that have been implicated in various neurological disorders such as epilepsy and the detection criteria do not fully describe the nature of the waveform but serve as a means to detect them in an automated way in an in silico model with mostly consistent action potentials [8, 9]. For both IISs and PDSs the frequency and mean duration are extracted. The other two variables that depict information regarding waveform shape are AUC and total line length of the voltage trace, which have also been found to express important information about the nature of neuronal signals in epilepsy such as signal variation and extremity [35, 36]. For each of these neuron specific variables, the mean, maximum, standard deviation, mean spatial gradient, maximum spatial gradient, and spatial gradient standard deviation were extracted for a total of 36 summary statistics.

The functional connectivity as determined by the TE has been related to a number of neurological disorders, including epilepsy [7, 37, 38]. The TE can be interpreted as a characterization of the amount of information one can derive about one time series from another time series. It is calculated by taking the logarithm of the ratio of the probability of observing a value at a particular time t in time series A given the past of time series A and the past of time series B to just that given the past of time series A, all weighted by the probability of observing the value given the past of time series A and the past of time series B and summing for each time point. If time series B provides useful predictive information about time series A, it logically follows that the two are functionally connected; the behavior of B affects the behavior of A. See Vicente et al. [37] for more information about how the Transfer Shannon entropy is calculated.

**Fig. 3:**
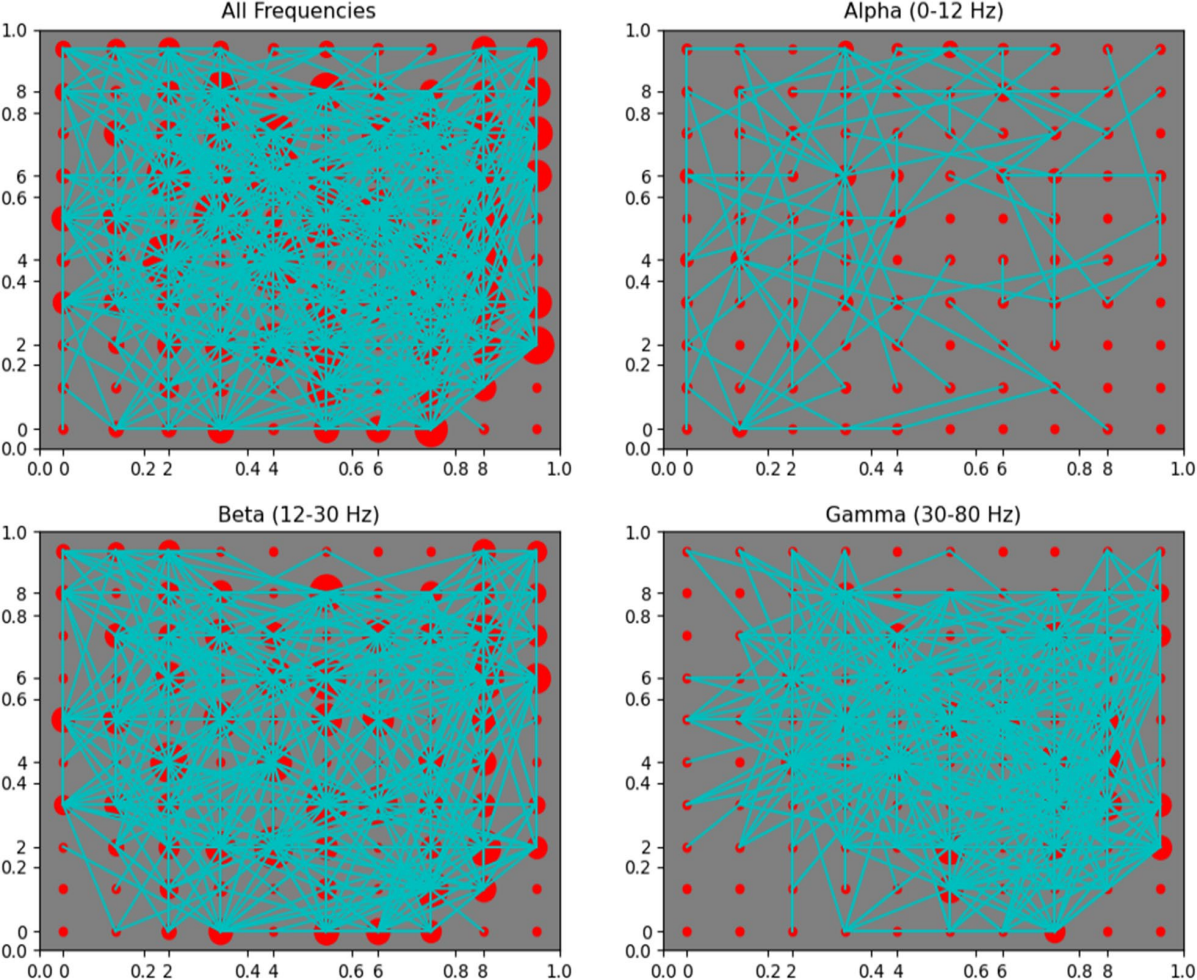
Functional Connectivity Plot. Exemplar network of functional connections of healthy neurons modeled on a 2D array as determined by the Transfer Shannon Entropy method. Blue lines indicate connections and red node size scales with number of connections

For each pair of neurons this was calculated in both directions, and if the TE was greater than 5e−5, the neuron was said to be functionally connected to the other. This was optimized to most closely correspond to a theoretical graphical mapping of neurons modeled to be physically connected with a separation of less than two nodes based on careful visual inspection of multiple models. Connections were categorized based on the origin node frequency band in the following ranges: Alpha (0–12.5 Hz), Beta (12.5–30 Hz), Gamma (30–80 Hz), and all frequencies. For each of the four frequency ranges, the mean number of connections for each neuron, maximum number of connections, total number of connections, standard deviation of the number of connections, mean number of connections spatial gradient, maximum number of connections spatial gradient, and the standard deviation of the spatial gradient of the number of connections was calculated for a total of 28 summary statistics. The identified connections for an exemplar subject are shown in Fig. 3.

### Machine learning approaches

With the 92 separate EP variables, various ML approaches were assessed by their ability to discern the status of an in silico subject; that is, given a set of EP variables, determining if the subject is healthy, GoF, or LoF. In order to ensure the information provided to the ML algorithms was valuable to a specified extent, a t-test was performed for each variable between each pair of groups and a threshold p-value was used as an inclusion criterion for particular feature. This value was set at 0.02, which was set to optimize the performance of the ML algorithms; this decision is discussed further in **Results**. Other methods of feature election [39] were considered and investigated with the aforementioned results yielding best model performance.

**Fig. 4:**
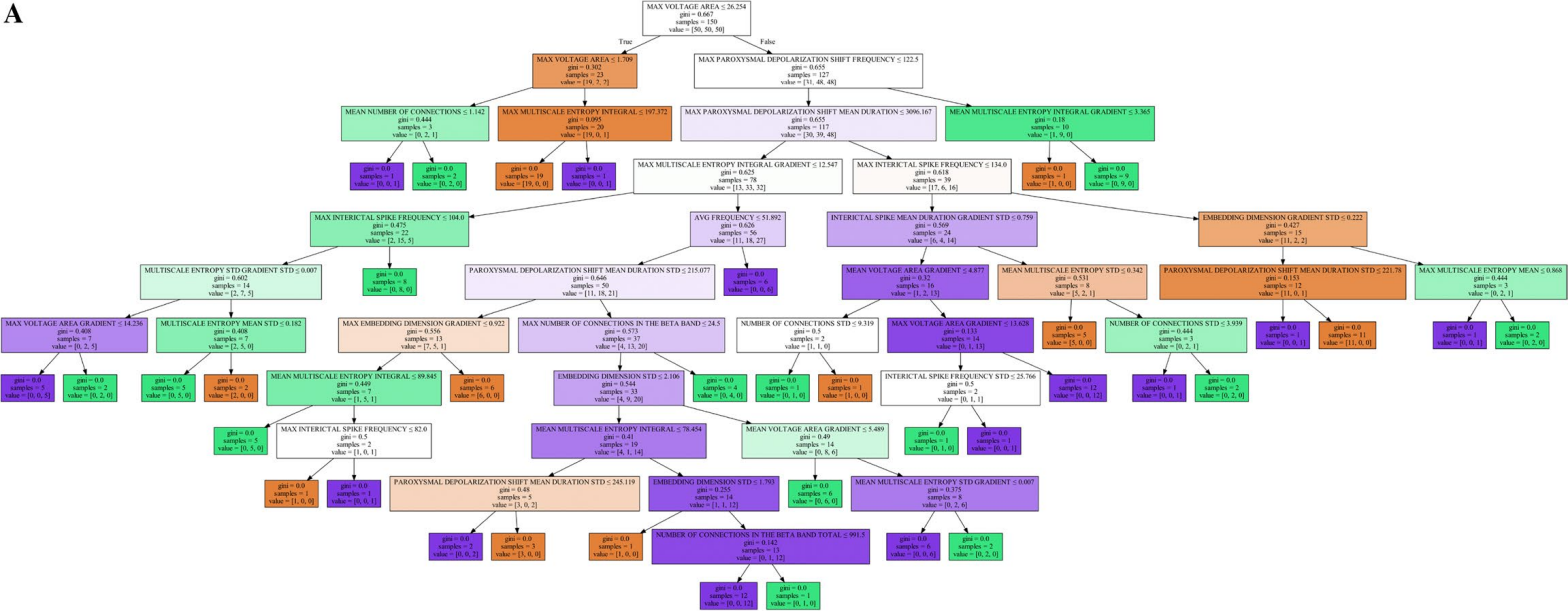

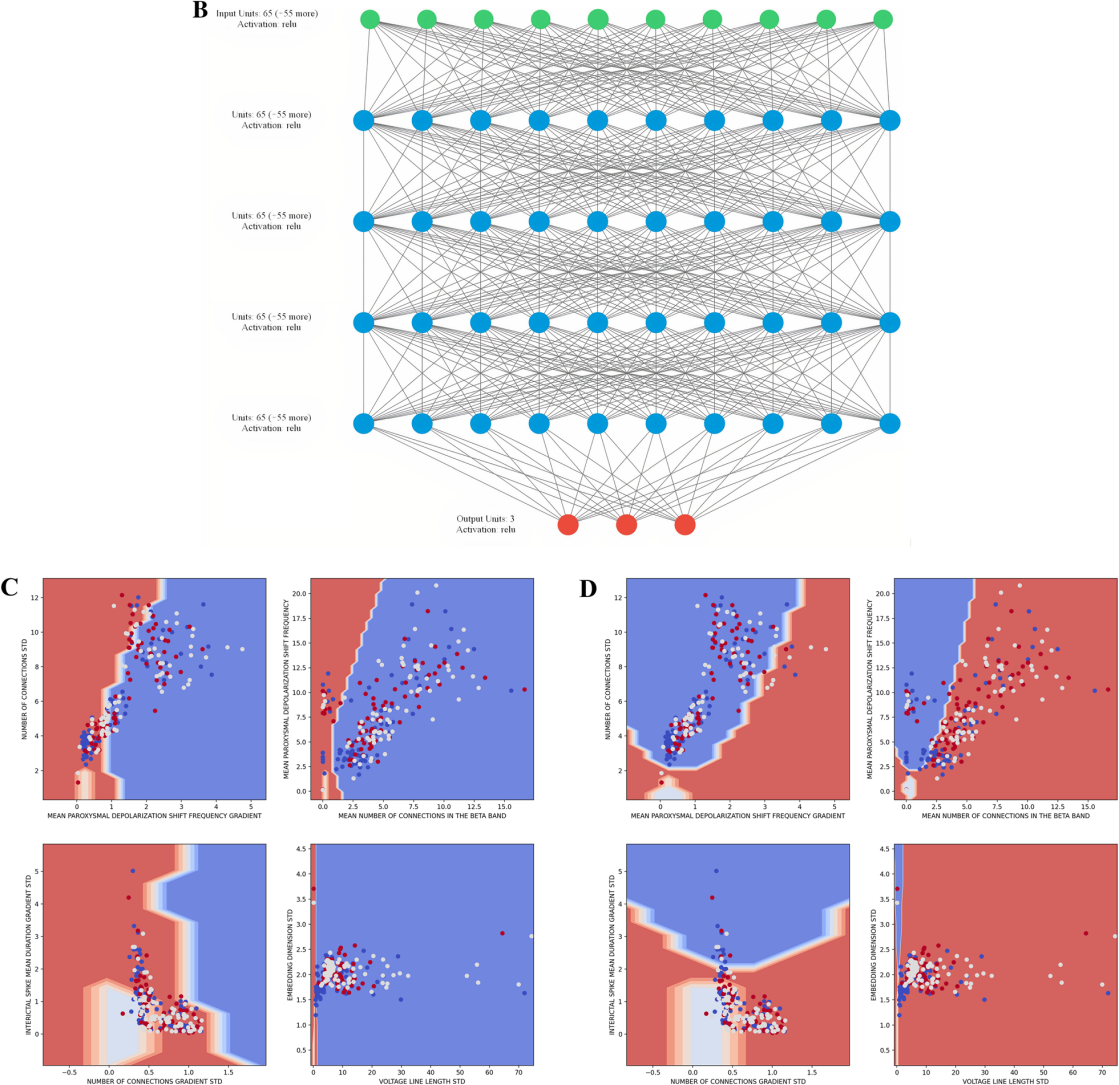
**A** Decision Tree. Exemplar Decision Tree classifier showing the decision at each stage indicated by the title of the box along with the gini and number of samples passed to said stage. Features of highest importance are located at the top of the decision tree and become less important with more branch points. The value corresponds to the number of subjects in the GoF, LoF, and healthy categories from the training set. **B** Neural Network structure. Sixty-five of the 92 EP features were found to meet the significance threshold to serve as input features. **C, D** SVM and GNB Graphical Depiction. Graphical depiction of 4 exemplar 2D projections of the higher dimensional space in which the SVM (**C**) and GNB (**D**) make delineations. The four images were chosen as examples that clearly show delineations between the classes as well as a sufficient number of data points. The color of the space indicates the category that a point lying in said space would belong to, given a z-score of 0 for the remaining variables. Since this is a projection of all other dimensions onto 2D space and not all other points have a z-score of 0 for all other EP variables, the dots do not match the space they are in. Red corresponds to GoF, white corresponds to Healthy, blue corresponds to LoF

Five ML approached were evaluated: a Neural Network (NN), a Support Vector Machine (SVM), a Gaussian Naïve Bayes Classifier (GNB), Decision Tree (DT), and Gradient Boosting Decision Tree (GBDT). Before use in the ML algorithms, the input variables were converted to z-scores to optimize the performance of ML methods affected by variable groups in different ranges. The NN was implemented with the Keras library with the TensorFlow backend and consisted of an input layer with a one-to-one correspondence of neurons with the input variables, followed by 3 identical hidden layers with rectified linear activation functions, and an output layer of three nodes with SoftMax activation. This structure is depicted in Fig. 4B. The activation functions and layer structure were chosen to minimize vanishing gradient and optimize performance. The SoftMax activation was chosen for the output such that each of the three nodes describe a probability that the given inputs are derived from each of the three model categories. With this type of output, the amenable sparse categorical cross entropy loss function was used and optimized with the ADAM optimization algorithm performed with 800 epochs at a batch size of 80. The remaining ML algorithms were implemented with the SciKit Learn. The Serial Vector machine regularization parameter was found to result in a best performing model at 0.2 with a radial basis function (RBF) kernel using the Grid Search library. All other parameters were found to optimize performance at their default values set by the SciKit Learn library for the SVM, GNB, DT, and GBDT. Each ML algorithm was trained with 50 models from each category and tested with the remaining 78 evenly distributed models and models were serialized based on meeting performance thresholds. Overall accuracy, true positive rates (TPRs), false positive rates (FPRs), and F1-scores were calculated for each class and ML method. A receiver operating characteristic (ROC) curve of best fit was found from the TPRs and FPRs of each class and method and integrated using the Simpson’s method to obtain AUC values. A graphical depiction of exemplar machine learning models is shown in Fig. 4A–D.

## Results

It has been noted that the significance threshold for including a variable in a ML model was a p-value of 0.02; this was chosen based on trial and error to optimize the performance of the ML algorithms. This is a better approach than making a multiple comparisons correction to the arbitrarily agreed upon 0.05 because the threshold for widespread acceptance of a result and probability of providing valuable information to differentiate groups are fundamentally different questions. Given that 92 variables were analyzed, the statistical correction for acceptance of results needs to be appropriate. It is clear that many of the 92 variables are intimately related and thus treating them as totally independent is irresponsible. For example, if the mean of a variable is found to be statistically significant between groups, one would expect the maximum and other derived quantities of the same metric to be different between the groups. Thus, treating these as 92 separate statistical tests would be overcorrecting. It is more reasonable to look at each category as something with mostly orthogonal information and use a correction closer to that of using 8 tests. However, even given the fact that each of the 8 categories are not completely orthogonal, it is also evident that each of the derived quantities are not completely dependent on the quantities in which they are derived from, especially those derived from the spatial gradients. Therefore, the authors agreed that a reasonable correction would be closer to that of 16 comparisons, leading to α = 0.003125. The results of the t-tests found 65 relevant features and are outlined in Additional file 1: Table S1.

**Fig. 5:**
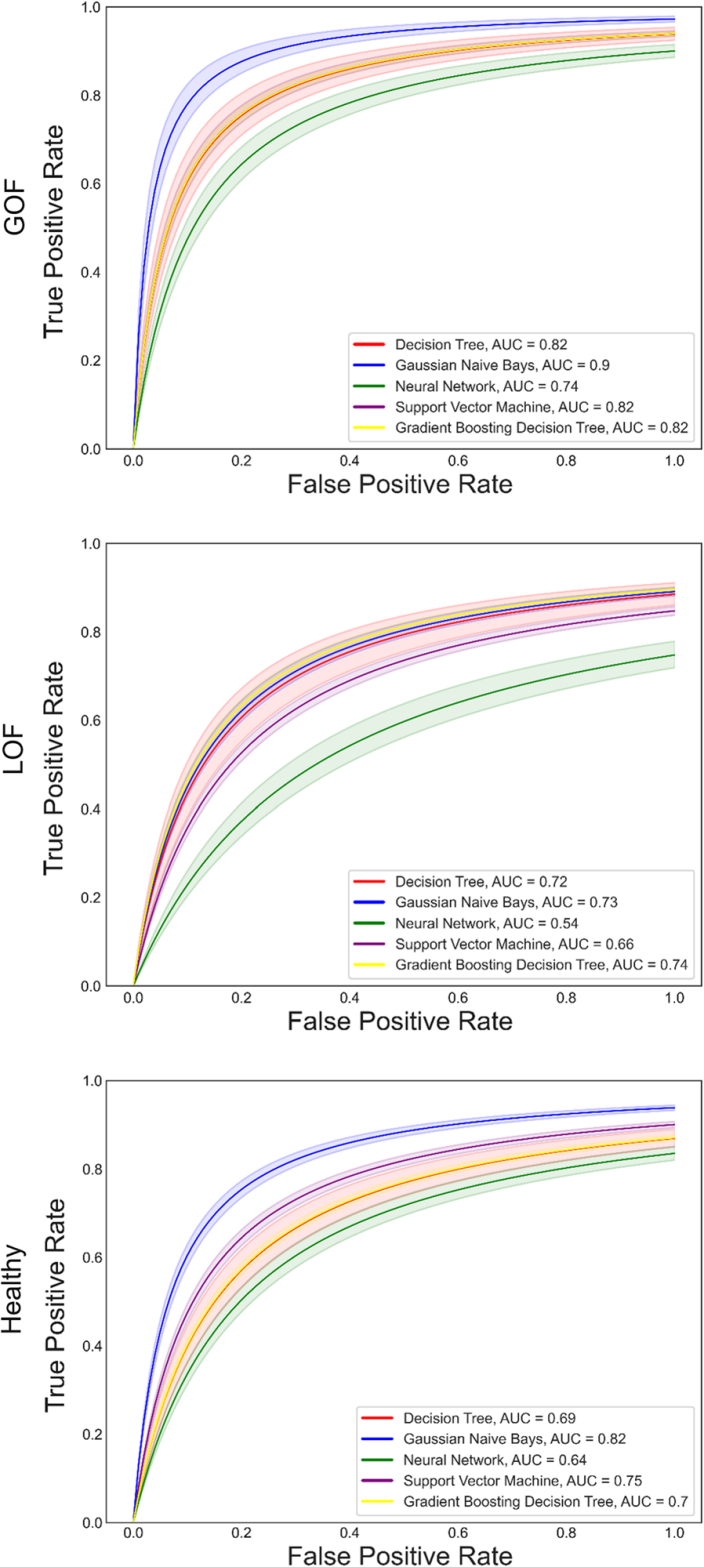
ROC Curves and AUC values. ROC curves were obtained by optimizing fit to the raw FPR and TPR data from serialized models and AUC values were obtained by integrating with Simpson’s method. The translucent border indicates the 99% confidence interval of the ROC curve of best fit

The performance of the Machine Learning models varied little within serialization of single models, but more between models. GNB performed best overall with an average accuracy of 65.98% which can be brought up to 70.51%, likely because it was far better at identifying Healthy (Accuracy = 83.19%, F1 = 0.67, AUC = 0.82) and GoF (Accuracy = 68.88%, F1 = 0.75, AUC = 0.9) subjects than the other models as indicated by all performance metrics, despite its worse overall performance in identifying LoF models. The best performing model for the LoF subjects was the Gradient Boosting Decision Tree with an average accuracy of 62.15% up to 76.92% (F1 = 0.61 and AUC = 0.74). The performance metrics for each model are summarized in Table 2 and AUC values are shown in Fig. 5.

**Table 2:** Summary of the relative performance of each model in the percent of testing subjects classified correctly indicated by mean (standard deviation)—max

	Overall	Healthy	GoF	LoF
*Decision tree*				
Accuracy	62.05 (1.54)–65.38	60.0 (6.92)–69.23	70.77 (8.8)–80.77	55.38 (9.3)–73.08
True Positive Rate		0.6 (0.07)–0.69	0.71 (0.09)–0.81	0.56 (0.09)–0.73
False Positive Rate		0.23 (0.05)–0.31	0.17 (0.05)–0.25	0.17 (0.05)–0.25
F1		0.58 (0.04)–0.64	0.69 (0.04)–0.76	0.58 (0.06)–0.7
*Gaussian Naive Bayes*				
Accuracy	65.98 (2.07)–70.51	83.19 (6.1)–100.0	68.88 (5.65)–80.77	45.86 (6.85)–61.54
True Positive Rate		0.83 (0.06)–1.0	0.69 (0.06)–0.81	0.46 (0.07)–0.62
False Positive Rate		0.33 (0.06)–0.48	0.07 (0.04)–0.17	0.11 (0.04)–0.23
F1		0.67 (0.03)–0.75	0.75 (0.04)–0.83	0.55 (0.05)–0.62
*Neural network*				
Accuracy	53.2 (1.5)–57.69	53.85 (7.45)–65.38	61.78 (7.38)–73.08	43.99 (9.42)–57.69
True Positive Rate		0.54 (0.08)–0.65	0.62 (0.07)–0.73	0.44 (0.09)–0.58
False Positive Rate		0.24 (0.05)–0.33	0.19 (0.06)–0.37	0.27 (0.06)–0.37
F1		0.53 (0.04)–0.59	0.62 (0.04)–0.67	0.44 (0.06)–0.53
*Support vector machine*				
Accuracy	60.34 (2.66)–65.38	72.46 (10.18)–92.31	60.18 (7.3)–76.92	48.37 (12.3)–73.08
True Positive Rate		0.72 (0.1)–0.92	0.6 (0.07)–0.77	0.48 (0.12)–0.73
False Positive Rate		0.31 (0.09)–0.58	0.11 (0.04)–0.19	0.18 (0.07)–0.33
F1		0.62 (0.04)–0.71	0.66 (0.06)–0.77	0.52 (0.08)–0.67
*Gradient boosting decision tree*				
Accuracy	62.55 (1.23)–66.67	59.16 (7.89)–76.92	66.32 (6.59)–80.77	62.15 (7.28)–76.92
True Positive Rate		0.59 (0.08)–0.77	0.66 (0.06)–0.81	0.62 (0.07)–0.77
False Positive Rate		0.22 (0.05)–0.35	0.14 (0.04)–0.25	0.2 (0.05)–0.31
F1		0.58 (0.04)–0.68	0.68 (0.04)–0.79	0.61 (0.05)–0.73

## Discussion and conclusions

Overall, the ML algorithms performed exceptionally well given only 1 s of EP data, with a maximum percent correct of 70.51%. Predictive power is further improved further by taking the best performing ML models from each category and having each of them make an independent prediction. For each model that predicted a given category, multiply each of their probabilities of being wrong (1—probability correct in category) giving the total probability all the predictions are wrong. Perform this for each category to derive a probability that the subject does not belong to every category. If none of the models predict a particular category, the probability that the given subject is not of that category is 1. Obviously, the lowest probability is the chosen prediction. Using this strategy, the current accuracy was brought to 74.14%. The largest obstacle to obtaining higher accuracy was the LoF models, however the GBDT model performs somewhat well here at average of 62.15% accuracy which can be brought up to 76.92% (F1 = 0.61, AUC = 0.74). The 20% decrease in inhibitory neuronal excitability had a very subtle effect on the overall subject model, and with only one second of EP data, differentiating them from healthy subjects was sometimes elusive to the ML algorithms. Due to this, models that are highly adept at classifying Healthy patients typically perform worse at classifying LoF patients and vice-versa. This results in a considerably higher class accuracy standard deviation than overall accuracy as the performances offset each other. Much of the current space in using ML to classify disease state from solely EP is assessing cardiac arrhythmias [14, 15, 40] which present with a number of simplifications compared to assessing neuronal pathologies. These include the ease of obtaining and accessibility of electrocardiograms (ECGs) compared to Electroencephalograms (EEGs) or MEA recordings of isogenic neurons as well as the significantly higher quality signal obtained from ECGs compared to the methods of recording neuronal signals. Finally, arrhythmias are very well defined and can be quickly identified by inspection whereas most of the EP perturbations that manifest as a result of neuropsychiatric pathologies are not well established or consistent. Despite these challenges, the classification metrics reach comparable overall classification levels as many of these methods with some models reaching 90–100% accuracy and 0.8–0.9 ROC AUC values for certain classes. Moreover, in addition to the challenges already present, this model simultaneously classifies between three states whereas most current research has focused on implementing models aimed at binary classification. The only related study the authors have found in the neuronal space was able to achieve a maximum accuracy of 93.1% with a maximum ROC AUC of 0.92 at binary classification [16] which is comparable to the best performing multiclass classification models in this study. This indicates the current study presents a considerable advance toward the direction of utilizing electrophysiological data to predict clinically valuable metrics due to the fact that ultimately, in this application, the classification must fundamentally not be limited to binary decisions. Clearly, more investigation is needed to bring this methodology to clinical applications as the potential number of patient classes may far exceed three. By replicating the present study in vitro significantly more model complexity can be observed and on much larger time scales. The resultant increase in information collection could significantly improve model bias in currently difficult to distinguish categories such as LoF and Healthy classes and augment model reliability, moreover by incorporating other measurable in vitro (-omic) biomarker classes it is expected that the performance of these methods has the potential to achieve excellent metrics with limited variance even with significantly expanded classification categories.

The subject models recapitulated in vitro MEA recordings quite well. All models showed classic Hodgkin-Huxley waveform shapes with occasional PDS and IIS, as well as time varying frequency and a notable graphical relationship between physically modeled and functional connectivity. There were a number of simplifications to the in silico model compared to in vitro models; notably, the lack of noise and well defined, consistent behavior that is controlled by parameters. This presents as the most notable limitation of the study and thus the conclusions need further validation using in vitro recordings of cultured neurons with known pathogenic mutation statuses. In vitro recording will likely have some noise despite consistent progress in noise filtering circuits, which may alter some of the variables. Moreover, the full complexity of neuronal cultures was not modeled here, but any number of the dozens of small factors such as temperature fluctuations, cell media used, and cell line specific genetics, among others, may have an impact on the extracted variables. Considering this, the results of this in silico study strongly encourage the implementation of an in vitro study in which these variables are in effect. Other limitations include sample size and duration of simulation, primarily due to computational and storage resources as well as a lack of benefit in an in silico context in regard to the increased signal homogeneity compared to in vitro recordings.

In addition to EP data, more information about the patient can help make predictions, such as demographic information, and certain genomic, transcriptomic, or other -omic information. Clearly, not all of this information is mutually exclusive, but this study has shown that sufficiently optimized ML algorithms are able to account for this on large scales with high accuracy. An in vitro study would have the additional benefit of using treatment response directly as an outcome, thus allowing for the genomic mutation status which categorizes a patient into a particular type of epilepsy to also be used as an input to the ML models.

In addition to the in vitro extension to this study, the results of the statistical analysis on the EP data encourage independent explorations into the utility of these variables as biomarkers for genetic epilepsy and verification of different manifestations of these biomarkers in vitro (MEA) and in vivo (EEG).

## Supplementary Information

**Additional file 1. Table S1.** Table of P values for each comparison used for feature selection among all extracted EP variables.

## Data Availability

Data from an exemplar model and the code underlying the in silico models, machine learning models, and variable extraction are available at https://github.com/gavinkress/Utilizing-Machine-Learning-Algorithms-to-Predict-Subject-Genetic-Mutation-Class-from-In-Silico-Model. Remaining individually generated models are available from gkress@usc.edu.

## References

[1] Engel J (2014). Approaches to refractory epilepsy. Ann Indian Acad Neurol.

[2] Begley CE, Famulari M, Annegers JF, Lairson DR, Reynolds TF, Coan S (2000). The cost of epilepsy in the United States: an estimate from population-based clinical and survey data. Epilepsia.

[3] Perucca P, Gilliam FG (2005). Adverse effects of antiepileptic drugs. Acta Neurol Scand Suppl.

[4] Perucca P, Carter J, Vahle V, Gilliam FG. Adverse antiepileptic drug effects Toward a clinically and neurobiologically relevant taxonomy [Internet]. 2009. Available from: www.neurology.org. 10.1212/01.wnl.0000345667.45642.61 PMC2677485 19349601

[5] Balestrini S, Sisodiya SM. Pharmacogenomics in epilepsy . Neurosci Lett [Internet]. 2018 [cited 2021 Sep 25];677:27–39. Available from: https://reader.elsevier.com/reader/sd/pii/S0304394017300241?token=0F2BBF1ADC5A4213519A46AAF1C1E4387988B866E4431609DAC24423CA851EE45397B98322DAAD182F3A7151546F61CA&originRegion=us-east-1&originCreation=20210927040608. 10.1016/j.neulet.2017.01.014 PMC5846849 28082152

[6] Amatya DN, Linker SB, Mendes APD, Santos R, Erikson G, Shokhirev MN (2019). Dynamical electrical complexity is reduced during neuronal differentiation in autism spectrum disorder. Stem Cell Rep.

[7] Douw L, van Dellen E, de Groot M, Heimans JJ, Klein M, Stam CJ, Reijneveld JC (2010). Epilepsy is related to theta band brain connectivity and network topology in brain tumor patients. BMC Neurosci.

[8] Staley KJ, Dudek FE (2006). Interictal spikes and epileptogenesis. Epilepsy Curr..

[9] Kubista H, Boehm S, Hotka M (2019). The paroxysmal depolarization shift: reconsidering its role in epilepsy, epileptogenesis and beyond. Int J Mol Sci.

[10] Kim Y, Rim YA, Yi H, Park N, Park SH, Ju JH. The generation of human induced pluripotent stem cells from blood cells: an efficient protocol using serial plating of reprogrammed cells by centrifugation. Stem Cells Int. 2016;2016. 10.1155/2016/1329459 PMC4989082 27579041

[11] Sharma A, Mücke M, Seidman CE (2018). Human induced pluripotent stem cell production and expansion from blood using a non-integrating viral reprogramming vector. Curr Protoc Mol Biol.

[12] Shi Y, Kirwan P, Livesey FJ (2012). Directed differentiation of human pluripotent stem cells to cerebral cortex neurons and neural networks. Nat Protoc.

[13] Gunhanlar N, Shpak G, van der Kroeg M, Gouty-Colomer LA, Munshi ST, Lendemeijer B (2018). A simplified protocol for differentiation of electrophysiologically mature neuronal networks from human induced pluripotent stem cells. Mol Psychiatry.

[14] Jurkiewicz J, Kroboth S, Zlochiver V, Hinow P (2021). Automated feature extraction from large cardiac electrophysiological data sets. J Electrocardiol.

[15] Zolotarev AM, Hansen BJ, Ivanova EA, Helfrich KM, Li N, Janssen PML (2020). Optical mapping-validated machine learning improves atrial fibrillation driver detection by multi-electrode mapping. Circ Arrhythm Electrophysiol.

[16] Levi R, Valderhaug VD, Castelbuono S, Sandvig A, Sandvig I, Barbieri R (2021). Bayesian supervised machine learning classification of neural networks with pathological perturbations. Biomed Phys Eng Express..

[17] Stimberg M, Goodman DFM, Brette R, de Pittà M (2017). Modeling neuron–glia interactions with the Brian 2 simulator. bioRxiv..

[18] Kaplan DI, Isom LL, Petrou S (2016). Role of sodium channels in epilepsy. Cold Spring Harb Perspect Med.

[19] Hodgkin AL, Huxley AF (1952). A quantitative description of membrane current and its application to conduction and excitation in nerve. J Physiol.

[20] Gulyá AI, Megías M, Emri Z, Freund TF. Total number and ratio of excitatory and inhibitory synapses converging onto single interneurons of different types in the CA1 area of the rat hippocampus. 1999; Available from: http://rsb.info.nih.gov/nih-image/. 10.1523/JNEUROSCI.19-22-10082.1999 PMC6782984 10559416

[21] Pastore VP, Massobrio P, Godjoski A, Martinoia S (2018). Identification of excitatory-inhibitory links and network topology in large-scale neuronal assemblies from multi-electrode recordings. PLoS Comput Biol.

[22] Tsodyks M (2005). Activity-dependent transmission in neocortical synapses. Les Houches Summer School Proc.

[23] Tsodyks M, Pawelzik K, Markram H. Communicated by laurence abbott neural networks with dynamic synapses. 10.1162/089976698300017502 9573407

[24] Vogt K (2015). Diversity in GABAergic signaling. Adv Pharmacol.

[25] Carlson BM. Tissues. The human body. 2019;27–63. Available from: https://linkinghub.elsevier.com/retrieve/pii/B9780128042540000028.

[26] Levick JR. Cardiac excitation and contraction. An Introduction to Cardiovascular Physiology. 1991;23–44.

[27] Traeger KA, Wen SF (2008). Pathophysiology of potassium metabolism. Pathophysiol Kidney Dis Hypertens.

[28] Zaza A (2000). The cardiac action potential. Introduction Cardiac Electrophysiol.

[29] Kobayashi T, Tohse N, Yokoshiki H, Sperelakis N. Developmental changes in ion channels. Cell Physiology Source Book. 2012;453–73.

[30] Masocha W (2016). Gene expression profile of sodium channel subunits in the anterior cingulate cortex during experimental paclitaxel-induced neuropathic pain in mice. PeerJ.

[31] Anwar A, Saleem S, Patel UK, Arumaithurai K, Malik P. Dravet syndrome: an overview. Cureus. 2019;11(6). 10.7759/cureus.5006 PMC6713249 31497436

[32] Kennel MB, Brown R, Abarbanel HDI (1992). Determining embedding dimension for phase-space reconstruction using a geometrical construction. Phys Rev A (Coll Park)..

[33] Wang DJJ, Jann K, Fan C, Qiao Y, Zang YF, Lu H (2018). Neurophysiological basis of multi-scale entropy of brain complexity and its relationship with functional connectivity. Front Neurosci.

[34] Jia Y, Gu H, Luo Q (2017). Sample entropy reveals an age-related reduction in the complexity of dynamic brain. Sci Rep.

[35] Liu S, Gurses C, Sha Z, Quach MM, Sencer A, Bebek N (2018). Stereotyped high-frequency oscillations discriminate seizure onset zones and critical functional cortex in focal epilepsy. Brain.

[36] Bergstrom RA, Choi JH, Manduca A, Shin HS, Worrell GA, Howe CL (2013). Automated identification of multiple seizure-related and interictal epileptiform event types in the EEG of mice. Sci Rep.

[37] Vicente R, Wibral M, Lindner M, Pipa G (2010). Transfer entropy—a model-free measure of effective connectivity for the neurosciences. J Comput Neurosci.

[38] Ursino M, Ricci G, Magosso E (2020). Transfer entropy as a measure of brain connectivity: a critical analysis with the help of neural mass models. Front Comput Neurosci.

[39] Pudjihartono N, Fadason T, Kempa-Liehr AW, O’Sullivan JM (2022). A review of feature selection methods for machine learning-based disease risk prediction. Front Bioinform.

[40] Alhusseini MI, Abuzaid F, Rogers AJ, Zaman JAB, Baykaner T, Clopton P (2020). Machine learning to classify intracardiac electrical patterns during atrial fibrillation: machine learning of atrial fibrillation. Circ Arrhythm Electrophysiol.

